# Dispositional mindfulness and BIS/BAS up-close: can the self-regulation of people be seen in the eyes?

**DOI:** 10.3389/fpsyg.2023.1217129

**Published:** 2023-08-10

**Authors:** Michaela Valachová, Elena Lisá

**Affiliations:** Institute of Applied Psychology, Faculty of Social and Economic Sciences, Comenius University in Bratislava, Bratislava, Slovakia

**Keywords:** dispositional mindfulness, behavioral approach system, behavioral inhibition system, self-regulation, iris structures

## Abstract

**Introduction:**

Pigmentation in animal models is related to behavioral regulation and development, suggesting that both may belong to the same biological system. However, such models are poorly documented in humans. The current study explored personality and group differences in self-regulation among healthy subjects and their specific eye structures (contraction furrows and pigment spots). Three objectives were proposed: to analyze statistical differences in dispositional mindfulness (DM), behavioral inhibition system (BIS), and behavioral approach system (BAS) among subjects with a specific iris type of contraction furrows and pigment spots.

**Methods:**

The study sample consisted of 194 university students. One month after taking photographs of their eyes, the students completed the online scales of DM, BIS, and BAS.

**Results:**

DM was negatively related to pigment spots (*r*_s_ = −0.193; *p* < 0.01). Cluster analysis of the iris structures converged at a four-cluster solution. The cluster types 2 (absence of pigment spots and contraction furrows extending 8/10 of iris circle or more) and 3 (one or more pigment spots and contraction furrows extending 8/10 of iris circle or more) significantly differed in DM with a small effect size (*F* = 3.37; *p* = 0.021; *η*^2^ = 0.051). Participants with contraction furrows (8/10 or more circle extent) and without pigment spots had a significantly higher DM than those with pigment spots. No significant differences existed among the iris types in BIS/BAS.

**Discussion:**

Future research directions are suggested.

## 1. Introduction

Examining the influence of biological determinants on human behavior is essential for understanding the connection between mind and body. This understanding is helpful for several reasons. For example, researchers may determine how physiology contributes to different human reactions or help individuals improve behavioral patterns by knowing inherited physiological predispositions such as self-regulation (Bell and Deater-Deckard, 2007) or drug response (Chaudhry et al., 2008).

Concerning physiology and personality, research findings support the neurobiological basis of temperamental traits such as neuroticism (DeYoung et al., 2010), sensitivity to rewards and punishment (BIS/BAS) (Ventura et al., 2019), or dispositional mindfulness (DM) (Taren et al., 2013). Specifically, temperamental and basic motivational-regulatory tendencies are associated with different brain regions (DeYoung et al., 2010; Taren et al., 2013; Rahman et al., 2014; Ventura et al., 2019). Personality dispositions depend on the volume of gray matter in specific brain areas (DeYoung et al., 2010). In summary, understanding the human brain may lead to a deeper understanding of human personality.

DM (Brown and Ryan, 2003) is the ability to be open to receptivity to the present moment (Brown and Ryan, 2003). BIS/BAS (Carver and White, 1994) determines the extent to which individuals perform optimally when exposed to an aversive stimulus (e.g., punishment) or appetitive stimulus (e.g., reward) (Gray and McNaughton, 2000). Both constructs are related to personality and self-regulation (Masicampo and Baumeister, 2007; Amodio et al., 2008; Brown et al., 2013). Although BIS/BAS is considered stable in time and hard to change (Takahashi et al., 2019), DM can be increased by regular exercise (Mothes et al., 2014). Moreover, DM can increase the efficacy of emotional regulation (Reese et al., 2015). DM with non-dysfunctional reward processing is a benefit to physical and psychological well-being (Carver and White, 1994; Baer, 2003; Taubitz et al., 2015). Moreover, DM has interventional potential (Baer, 2003; Takahashi et al., 2019). Fast identification of a person’s DM level may enable targeting and developing appropriate and effective self-regulation interventions.

Soh et al. (2021) recommend reviewing eye structures jointly as types (clusters). Concerning the connection between physiology and personality dispositions, through cluster analysis, Larsson et al. (2007) found that specific iris structures (e.g., contraction furrows) could differentiate between individuals scoring high and low on neuroticism. Although such results seem interesting and valuable, more credible research is required. Usually, evaluating DM and sensitivity to reward/punishment is based on self-reports or, exceptionally, on brain imaging techniques. The research question may arise whether we can detect dispositional tendencies to self-regulation using biological markers (such as iris structures) without exploring the brain directly.

Other specific iris structures, e.g., Fuchs’ crypts, pigment spots, and Wolfflin nodules, have been significantly associated with temperamental personality traits (Larsson et al., 2007), Down syndrome (Postolache and Parsa, 2018) or schizophrenia (Trixler and Tényi, 2017; Tian et al., 2022). No study has explored DM, the behavioral inhibition system (BIS), and the behavioral approach system (BAS) regarding the iris structures. Exploring physiological markers beyond the brain indicating a DM and BIS/BAS level may extend such scarce research.

The DM and BIS/BAS are activated in the dorsomedial prefrontal cortex (dmPFC) (Modinos et al., 2010; Eryilmaz et al., 2017), which is the brain area previously suggested to be associated with the iris/brain tissue loss hypothesis (tissue loss in the iris-hypoplasia is associated with tissue loss in brain structures) (Larsson et al., 2007). Furthermore, the BIS/BAS systems are biologically driven dispositions related to anatomical differences in brain volume (Ventura et al., 2019). Therefore, it seems plausible to consider the relevance of exploring the possible connections between these psychological constructs and specific iris markers. Can self-regulation be observed in the eyes?

Given the above, the current study explores whether specific iris structures (contraction furrows and pigment spots) indicate differences in DM and BIS/BAS in a non-clinical sample of university students.

### 1.1. Self-regulation

In the literature, the term self-regulation often refers to different concepts. The current study operationalizes self-regulation using two personality constructs, DM (Brown and Ryan, 2003) and BIS/BAS, derived from reinforcement sensitivity theory (RST; Gray, 1987).

Theories and empirical evidence suggest that personality development is a dynamic lifelong process biologically and environmentally determined during life (Kandler, 2012). RST theory explains how neurobiological mechanisms for behavioral regulation relate to personality. The BIS/BAS deals with individual differences in reward (BAS) and punishment processing (BIS) (Corr et al., 2013). The BIS controls the experience of anxiety, fear, or frustration in response to environmental cues and inhibits movement toward goals (Gray, 1987; Carver and White, 1994). The BAS controls appetitive motivation and feelings about impending rewards, such as happiness, craving, and impulsivity (Carver and White, 1994). These individual differences are visible on brain scans (DeYoung et al., 2010; Rahman et al., 2014; Ventura et al., 2019).

Research on the RST has revealed a solid biological basis for universal personality dispositions. Temperamental traits (e.g., neuroticism and impulsivity) originating from the Big Five model (McCrae and Costa, 2008) are closely related to the neurobehavioral systems of the BIS/BAS (Carver and White, 1994; Smits and Boeck, 2006). The biological basis of temperamental traits was presumed in the original research on iris structures (Larsson et al., 2007). The results showed that students with higher impulsivity had more contraction furrows in their irises.

DM is another concept related to self-regulation and emotional regulation, which vary with personality. DM predicts self-regulated behaviors and positive emotional states (Brown and Ryan, 2003). Individual differences in DM reflect differences in the recognition, detachment, and regulation of everyday experience (Modinos et al., 2010). A higher level of DM is associated with a better ability to differentiate emotional experiences, reflected in a more efficient ability to regulate emotional experiences (Hill and Updegraff, 2012). A higher DM is negatively related to neuroticism (Hanley, 2016). Effective and fast identification of one’s DM level by iris characteristics may help address psychological intervention strategies faster.

The research on associations between DM and BIS/BAS brings consistent findings. The BIS and BAS relate to mindfulness-based emotional experiences (Reese et al., 2015; Dolatyar and Walker, 2020). However, DM does not affect the change in BIS/BAS in the intervention programs (Takahashi et al., 2019). BAS impulsivity negatively correlates with the mindfulness dimension of awareness (du Rocher, 2022). A mindless response (the impulsive response of individuals who cannot regulate their behavior in the present moment) to rewards is not associated with awareness (Dolatyar and Walker, 2020). In other words, mindful responses (when individuals are fully aware of their actions) are related to their ability to regulate their impulses. At the neurobiological level, mindfulness is associated with the dorsal region of the brain (dmPFC), which is the same region related to the BIS/BAS (Modinos et al., 2010; Eryilmaz et al., 2017). Further, in the longitudinal study, Karl et al. (2021) found that mindfulness and BIS/BAS share developmental trajectories. Thus, the same biological system may determine variations in an activity that imply individual differences in mindfulness or the BIS/BAS.

### 1.2. The current study

The main goal is to explore whether iris structures (clustered in types) point to differences in the self-reported DM and BIS/BAS levels. DM and BIS/BAS predict self-regulation (Brown and Ryan, 2003) and personality traits such as neuroticism, extraversion (Carver and White, 1994; Smits and Boeck, 2006) and can be recognized within specific brain areas detected by brain scans (Modinos et al., 2010; Eryilmaz et al., 2017). These brain areas are considered important to personality and iris structures. Specific iris structures are related to personality traits (Larsson et al., 2007), Down syndrome (Postolache and Parsa, 2018), or mental disorders (Trixler and Tényi, 2017; Tian et al., 2022).

Various specific eye structures are moderate to highly heritable. They can be defined as biomarkers reflecting genetic differences among populations (Sturm and Larsson, 2009). Contraction furrows are produced by pupil contraction and dilation (Chua et al., 2017). The overall thickness and density of the iris play important roles in its formation (Larsson and Pedersen, 2004). Contraction furrows significantly correlate with thicker irises (Chua et al., 2017). This iris structure varies across populations. More specifically, East Asians have a significantly lower furrow grade than either South Asians or Europeans (Edwards et al., 2016) because of the different thicknesses and pigmentation in the eyes (dark eyes).

Some iris structures are inter-correlated because of their shared genetic bases (Sturm and Larsson, 2009). Thinning contraction furrows relate positively to iris pigment spots (Larsson and Pedersen, 2004; Sturm and Larsson, 2009; Edwards et al., 2016). The pigment spots are genetically determined (Sulem et al., 2008) as small regions of hyperpigmentation in the anterior border layer. They may be superficial (freckles) or distort the underlying stromal layer (nevi) (Harbour et al., 2004; Chua et al., 2017). More than half of the Europeans (57.9%) show some degree of iridial spotting compared to 22.1% of East Asians and 16.7% of South Asians. Again, these differences are due to the higher occurrence of dark eyes, in which fewer spots are visible because of hyperpigmentation (Edwards et al., 2016).

Some researchers have suggested that pigment spots could be associated with personality because the development of pigment spots is partially regulated by neurotransmitters produced by the autonomic nervous system (Hu, 2000; Hu et al., 2000). Pigment spots arise (in the skin) because of ultraviolet light (UV) or stress and cortisol release. The increase of dopamine (e.g., by practicing yoga) may contrast cortisol level and consequently prevent the formation of pigment spots (Yilmaz, 2023). The idea that morphology is connected to regulation and behavior is not new to non-human animal research. An experiment on fox domestication (Trut et al., 2004) showed that the same biological system might cause changes in fur pigmentation (in tamed foxes) and behavioral regulation. Spotted pigmentation occurs even in flowers within self-organizing activator-inhibitor systems caused by specific proteins (Ding et al., 2020). In humans, mental conditions such as schizophrenia, which is characterized by severe cognitive impairment and emotional dysregulation, and low level of mindfulness (Tabak et al., 2015), are related to a higher level of pigment spots in the eyes (Trixler and Tényi, 2017; Tian et al., 2022). There are also indications that the same genetic system influences neuronal development and iris formation (Larsson et al., 2011). Beyond pigment spots, another iris structure – contraction furrows, are related to impulsivity and differentiate between individuals who score high and low on the neuroticism domain (Larsson et al., 2007), which reflects a person’s ability to regulate negative affect (McCrae and Costa, 2008).

Based on all above, we propose research objectives (RO) that furrows and pigment spots will differentiate individuals who will score high and low on the DM (RO1), BIS (RO2), and BAS (RO3) because of their empirical connection to the personality traits (impulsivity, neuroticism) (Larsson et al., 2007) and self-regulation (Hu et al., 2000).

RO1: to analyze a statistical difference in dispositional mindfulness (DM) among participants with specific types of irises (contraction furrows and pigment spots).

RO2: to analyze a statistical difference in behavioral inhibition (BIS) among participants with specific types of irises (contraction furrows and pigment spots).

RO3: to analyze a statistical difference in behavioral activation (BAS) among participants with specific types of irises (contraction furrows and pigment spots).

## 2. Methods

### 2.1. Participants

The research sample consisted of 194 university students of psychology, law, philosophy, and economy (30% men) with 21,6 years of age on average (SD = 3,4), ranging from 18 to 36. Power analysis with G*power for one-way ANOVA (Effect size *f* = 0.25; *α* err prob. = 0.05; Power (1 − *β* err prob) = 0.80; the number of groups = 4) calculated total sample size of *N* = 180. The achieved sample size *N* = 194 was sufficient by its size.

From the original 257 participants, 194 passed the inclusion criteria: age 18 and more, without a diagnosis of epilepsy or mental condition, and with correct answers to the control attention questions. Sixty-three participants were excluded from the analysis because of not meeting these criteria.

Quantitative research was conducted with a time-lagged cross-sectional, comparative, and correlational design. The study was divided into two phases from October to December 2021. In Phase 1, students were contacted to enter the lab and take macro photos. They signed a written informed consent form agreeing to publish their eye photos in the research study. Participation was voluntary. In Phase 2, 1 month after taking photographs of their eyes, the students completed the online questionnaire with scales of DM, BIS, and BAS. The online questionnaire included informed consent and research scales in the order listed below. Three control questions were included in the questionnaire.

### 2.2. Research ethics

The Faculty of Social and Economic Sciences, Comenius University, Bratislava, approved the study (FSEV 823-4/2022 SD). All participants provided informed consent, voluntary provision of information, and the option to leave whenever they wanted without any consequences. The participants agreed to aggregated data analysis for the research study. There were no foreseeable intended or unintended adverse effects on the participants. The standard procedure described in several other published studies was used (Larsson et al., 2007; Edwards et al., 2016; Tian et al., 2022) without the supervision of an ophthalmologist. Participants made no complaints about difficulties caused by photographing their eyes. A professional photographer captured photographs. The photographer ensured the highest possible comfort for the participants when photographing their eyes. The entire procedure took 5 min. Preparation for the photo shoot took almost all this time, and the photo shoot itself took place within seconds. Participants whose eyes were placed in the manuscript agreed to the publication of their eye macro photographs in written form. All methods followed the Personal Data Protection Act No. 18/2018, Coll., and Internal University Regulation Nb. 23/2016.

### 2.3. Measurements

*BIS/BAS scale* (Carver and White, 1994) included 20 items and two dimensions of the behavioral aversive system scale (BIS), and three dimensions of behavioral approach systems scales (BAS). BIS included seven items and assessed sensitivity to punishment (e.g., “Criticism or scolding hurts me quite a bit”; *α* = 0.78; *ω* = 0.78). BAS included three facets: reward responsiveness (five items, e.g., “When good things happen to me, it affects me strongly”; *α* = 0.63; *ω* = 0.65), Drive (four items, e.g., “When I go after something, I use a “no holds barred” approach”; *α* = 0.84; *ω* = 0.84), Fun Seeking (four items, e.g., “I often act on the spur of the moment”; *α* = 0.77; *ω* = 0.77). The participants responded on a four-point Likert scale ranging from 1 (strongly disagree) to 4 (strongly agree), averaging 1 to 4. The model had an adequate data fit *X*^2^ (*N* = 257; *df* = 164) = 264.04; TLI = 0.979; CFI = 0.982; RMSEA = 0.049; *p* < 0.001; SRMR = 0.080.

*The Mindful Attention Awareness Scale* (Brown and Ryan, 2003) measured DM with 15 items. The scale was developed to measure the presence or absence of awareness of what is happening at a given moment (*α* = 0.79; *ω* = 0.80). Items were designed to reflect the experience of mindfulness in general and specific everyday situations, including variations in the awareness of thoughts, emotions, and physical states. Participants answered how often they have the experience described in each statement using a 6-point Likert scale (1 = almost always; 6 = almost never, e.g., “It seems I am “running on automatic,” without much awareness of what I’m doing”). The model had an adequate data fit: *X*^2^ (*N* = 257, *df* = 90) = 133.91, TLI = 0.984, CFI = 0.986, RMSEA = 0.044, *p* < 0.001; SRMR = 0.063.

*The iris characteristics* were photographed. Photographs were taken in a darkened room using a camera placed on a tripod. To prevent unwanted movements, the participants rested their heads on a high stool attached to a tripod while sitting on an adjustable chair. Canon 6D cameras with EF 100 mm f/2.8 L Macro lenses were used. Camera support made it very easy to capture eye pictures because the autofocus helped and could work much more accurately by having the camera centered on the iris and at the same distance from each iris. The aperture was set to f20, 1/80s shutter speed, and ISO-2000 to achieve optimal image sharpness. Therefore, Speedlite external flash was used. A small flashlight was attached to the flash, pointing straight into the iris. Pictures of both irises of the subjects were captured. The focal distance was set as 140 mm. A photographer used a pad on the stool to secure a fixed distance from the chins of the subjects. The resolution of the photos was 5,472 × 3,648 pixels with 300 pixels/inch in the CR2 RAW format. Adobe Photoshop software was used to adjust the contrast and brightness of the final images.

### 2.4. Procedure

The photos were downloaded into the rating sheet and prepared for scaling. Cut-off points for the iris structures were provided by Dr. Larsson, derived from the author of the original study (Larsson et al., 2003)contraction furrows were coded by 1 when extending less than ¼ circle, 2 when extending between 1/4 and 8/10, and 3 with contraction furrows more distinct, extending 8/10 of one circle or more. Pigment spots were coded as 1 (absence of pigment spots), 2 (one to four pigment spots), and 3 (five or more spots).

In the current study, two raters assessed the number of pigment spots and the extent of contraction furrows. The extent of furrows lower than ¼ of the eye circle was coded as 1, and the extent higher than 8/10 of the iris circle was coded as 2. The absence of pigment spots was coded as 1, and one to four pigment spots were coded as 2. The assessors reviewed photographs of the left iris. The ratings were performed in a room with subdued soft lighting. The assessors sat approximately 0.50 meters from the computer screen. The photos were presented at the magnification of 100%; the diameter of the iris was 180–190 mm for ratings of pigment spots and furrows. The scales’ inter-rater reliability was 0.91 (for furrows) to 0.97 (for pigment spots), according to Cohen’s kappa (Cohen, 1960). The photos which did not receive the same ratings from the two judges were omitted from the study.

Furrows and pigment spots on the iris were rated using quantitative and reproducible methods. The assessors’ judgments were scored on an ordinal/nominal scale. Photographs to be rated were randomly sorted into catalogs. After providing 100 ratings, the evaluators controlled for reliability. Discrepancies between the ratings were discussed with the test leader. Dr. Mats Larsson, the author of the scale, offered the original scales for this project.

### 2.5. Data analysis

Data were not normally distributed. CFA was applied to analyze the measurements’ structure, with a 95% confidence interval, 5,000 Bootstrap replications, and a DWLS estimator for ordinal variables (Li, 2016). Comparative Fit Index (CFI), Tucker-Lewis Index (TLI), the Root Mean Square Error of Approximation (RMSEA), and the Standardized Root Mean Squared Error (SRMR) were the estimation indexes for CFA. The cutoff values for CFI and TLI were adequate when >0.90 and good when >0.95 (Marsh et al., 2004), for RMSEA and SRMR adequate, when <0.08 and good when <0.05 (Hu and Bentler, 1999).

A two-step cluster analysis was conducted, with log-likelihood as the distance measure, with an automatically determined number of clusters through Schwarz’s Bayesian Criterion (BIC) in IBM SPSS. The eye structures were coded as 1 for low and 2 for high levels. ANOVA was used to analyze the differences among types in the self-regulation variables. The Eta squared value was calculated to estimate the effect size and Dunn’s *post hoc* tests for optimal testing of small subsets of pairs. The *post hoc* test was a follow-up to the Kruskal-Wallis test. Holm’s sequential Bonferroni correction was applied to correct for multiple comparison testing and avoid Type I errors (false-positive results) (Holm, 1979). Data were processed in IBM SPSS 22, MS Excel, JASP 0.16.3 (JASP Team, 2022), and G*Power 3.1.9.4.

The reflective measurement models were applied because the independent variables (iris structures) were expected to be inter-correlated with a shared latent factor – genetics (Larsson and Pedersen, 2004). Dependent variables (personality dispositions) reflect their own latent variables.

## 3. Results

### 3.1. Correlational analysis

Spearman correlations were analyzed for all variables due to the ordinary character of iris characteristics (Clatworthy et al., 2005; Soh et al., 2021). There was a small negative correlation between DM and pigment spots (*r*_s_ = −0.193; *p* < 0.01) and between DM and BAS fun seeking (*r*_s_ = −0.239; *p* < 0.001). The less mindful participants were, the more pigment spots they had. The less mindful participants were, the higher in BAS fun-seeking they were. All BAS dimensions correlated among themselves, which supports their belonging under the common construct of the behavioral activation system. BIS correlated negatively with BAS fun (*r*_s_ = −0.132; *p* < 0.01). There was no significant correlation between the iris characteristics and BIS/BAS scores (Table 1).

**Table 1:** Spearman’s correlations among analyzed variables.

	1	2	3	4	5	6	7
1. BIS	—						
2. BAS reward	0.119	—					
3. BAS drive	−0.073	0.530***	—				
4. BAS fun	−0.132*	0.347***	0.270***	—			
5. DM	−0.075	−0.057	0.028	−0.239***	—		
6. Furrows	0.008	−0.021	−0.030	−0.083	0.013	—	
7. Pigment spots	0.062	0.069	0.028	0.070	−0.193**	0.042	—

*p < 0.05, **p < 0.01, and ***p < 0.001.

### 3.2. Cluster analysis

**Figure 1:**
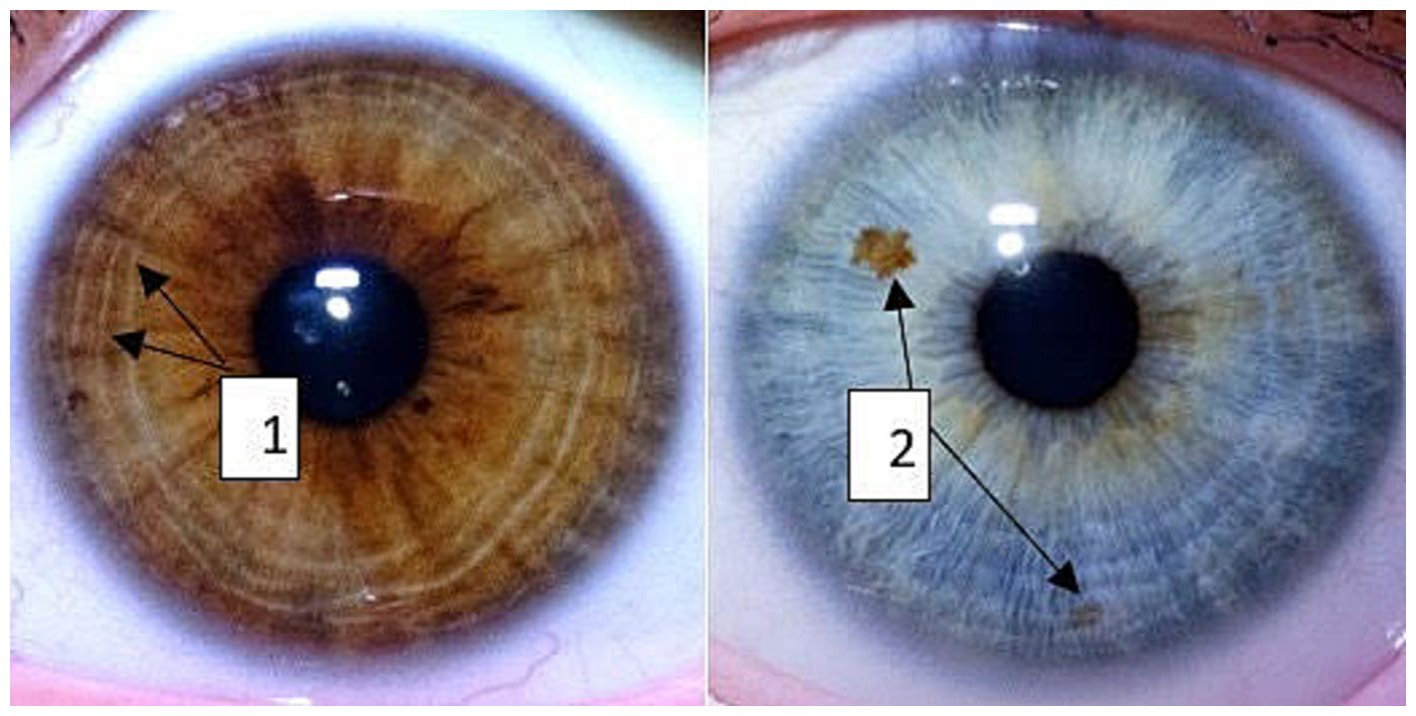
Contraction furrows (1) and pigment spots (2) as the iris’s characteristics.

The cluster analysis determined iris types with pigment spots and furrows. Examples of contraction furrows (marked as 1), and pigment spots (marked as 2) are shown in Figure 1. The iris characteristics were coded regarding their extent (furrows) or number (pigment spots).

The results of the two-step cluster analysis, with log-likelihood as the distance measure and number of clusters determined by Schwarz’s Bayesian Criterion (BIC), showed a four clusters solution. Cluster 1 had 19%, Cluster 2 29%, Cluster 3 21%, and Cluster 4 31% of the variance. The size ratio (largest to smallest cluster) was 1.62, meaning there was a balanced representation of participants in all clusters. The cluster quality (silhouette measure of cohesion and separation) was good, with an average silhouette of 1.00. It means that each of the clusters included specific cases of iris structure. It was not possible for some of the participants to belong, even hypothetically, to several clusters. Each participant belonged exclusively to one of the four identified clusters. The input and predictor importance of the variables for the results were 100% for pigment spots and furrows, meaning that information on both iris characteristics (furrows, pigment spots) counted 100% for cluster identification. Table 2 lists the structural values of each type.

**Table 2:** Values of iris structures for the four clusters/types.

	Furrows	Pigment spots
Cluster/Type 1 (*n* = 37)	1 (extending less than ¼ of circle)	2 (one to four pigment spots)
Cluster/Type 2 (*n* = 56)	2 (extending 8/10 of circle or more)	1 (absence of pigment spots)
Cluster/Type 3 (*n* = 41)	2 (extending 8/10 of circle or more)	2 (one to four pigment spots)
Cluster/Type 4 (*n* = 60)	1 (extending less than ¼ of circle)	1 (absence of pigment spots)

Ordinal values of furrow and pigment spots in the cells. For example, Cluster/Type 1 included people with furrows coded as 1 and pigment spots coded as 2.

There were 37 participants in cluster 1, with contraction furrows extending less than ¼ of the circle and with one to four pigment spots. There were 56 participants in cluster 2, with contraction furrows extending 8/10 of the circle or more and without pigment spots. There were 41 participants in cluster 3, with contraction furrows extending 8/10 of the circle or more and with one to four pigment spots. There were 60 participants in cluster 4, with contraction furrows extending less than ¼ of the circle and without pigment spots.

The extent of furrows lower than ¼ of the eye circle was coded as 1, and the extent higher than 8/10 of the iris circle was coded as 2. No participants had middle values between ¼ and 8%10 of circle extent. The absence of pigment spots was coded as 1, and one to four pigment spots were coded as 2. No participants had five or more pigment spots in their iris.

### 3.3. Differences in self-regulation among types

There were no significant differences among types in BIS (*F* = 0.49; *p* = 0.689; *η*^2^ = 0.008). There were no significant differences among types in BAS reward (*F* = 0.62; *p* = 0.606; *η*^2^ = 0.010). There were no significant differences among types in BAS drive (*F* = 0.64; *p* = 0.589; *η*^2^ = 0.010). There were no significant differences among types in BAS fun-seeking (*F* = 0.65; *p* = 0.587; *η*^2^ = 0.010). Students with the presence or absence of furrows and pigment spots did not differ in how they were behaviorally inhibited or activated.

There were significant differences among types in DM (*F* = 3.37; *p* = 0.021; *η*^2^ = 0.051). Types 2 and 3 differed significantly (*p* = 0.011; *p*_holm_ = 0.011). Type 2 had the highest (M = 3.92; SD = 0.73) and type 3 had the lowest (M = 3.47; SD = 0.84) DM. Specifically, type 2 was characterized by contraction furrows and the absence of pigment spots. Type 3 had both contraction furrows and pigment spots. Students who had irises with furrows but without pigment spots had the highest level of DM. Students who had furrows and pigment spots had the lowest level of DM. Having pigment spots or not having them alone is not enough for differences in DM if the participants did not have contraction furrows.

Table 3 displays the means and standard deviations for measured variables clustered in the iris types. DM was the highest in cluster 2 and the lowest in cluster 3. The difference between the highest and the lowest value in DM within clusters was 0.45. The difference between the highest and the lowest value in BAS fun and reward was 0.10, in BIS 0.12, and in BAS drive 0.19, indicating that the levels of BIS/BAS were quite similar in all of the iris clusters.

**Table 3:** Means and standard deviations for analyzed variables in the iris types.

Iris types	BIS		BAS fun seeking		BAS reward		BAS drive		Dispositional mindfulness	
	M	SD	M	SD	M	SD	M	SD	M	SD
1	3.17	0.45	3.38	0.50	3.72	0.26	3.13	0.58	3.62	0.62
2	3.07	0.59	3.26	0.58	3.62	0.39	3.08	0.64	3.92	0.72
3	3.14	0.49	3.20	0.60	3.62	0.41	2.94	0.70	3.47	0.83
4	3.05	0.59	3.30	0.61	3.64	0.34	3.05	0.64	3.76	0.68

**Figure 2:**
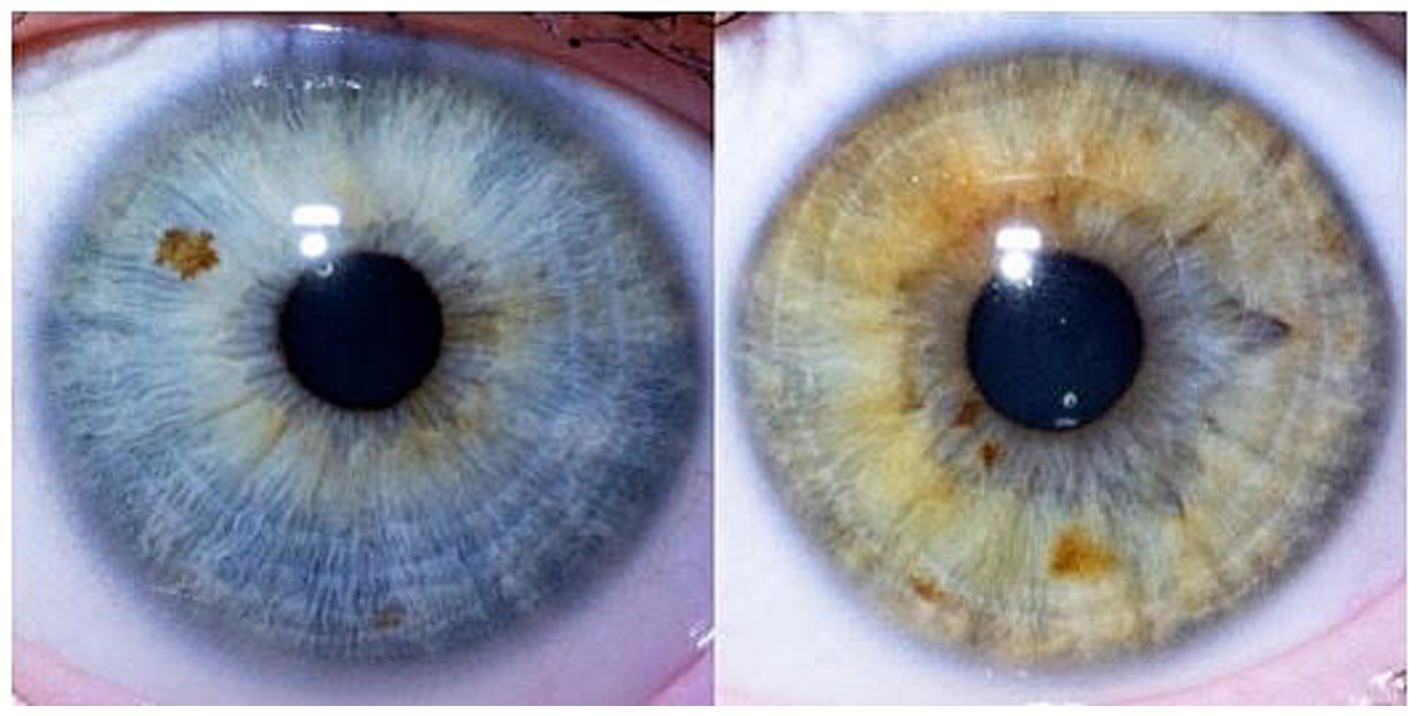
Typical cluster 3 – extended furrows, visible pigment spots.

Figure 2 displays a typical representative of cluster 3, with an iris of extended furrows and visible pigment spots.

**Figure 3:**
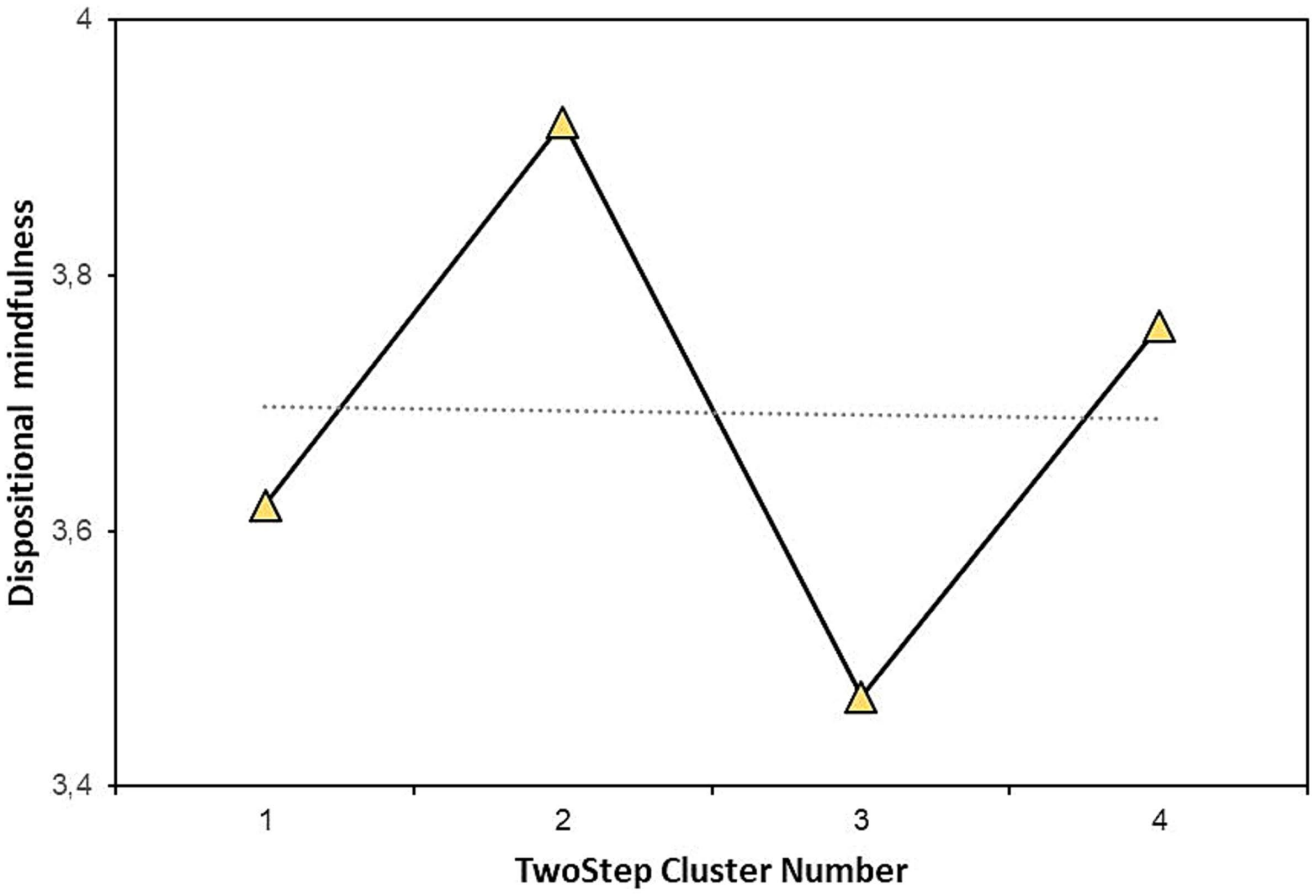
Means of dispositional mindfulness in the iris types.

Figure 3 shows the mean values of DM for all the types. It is visible that the biggest differences in DM were between types 2 and 3, the types with extended contraction furrows and with/without pigment spots. Types without contraction furrows did not differ among themselves or from other types.

## 4. Discussion

The study explored personality differences between participants with specific eye structures such as furrows and pigment spots. The main goal was to explore whether iris structures show differences in the self-reported DM and BIS/BAS levels. We stated three research objectives to analyze a statistical difference in DM, BIS, and BAS among types/clusters with a specific iris type of furrows and pigment spots. Although there were no published studies examining personality variables selected for the current study, the research objectives were built on empirical studies investigating iris structures in association with temperamental traits (Larsson et al., 2007) or mental conditions (Trixler and Tényi, 2017; Tian et al., 2022) associated with self-regulation issues.

The first research objective was to analyze the differences in DM among participants clustered in different iris types (based on the combination of pigment spots and contraction furrows). Type with pigment spots and contraction furrows had lower DM than type without pigment spots and with contraction furrows. The type with pigment spots and contraction furrows had the lowest DM, and the type with no pigment spots and with contraction furrows had the highest DM. Furrows with pigment spots may reflect differences in how people are open to receptivity to the present moment or how they tend to regulate everyday experience. The second and third research objectives aimed to analyze BIS/BAS levels in participants with different iris structures clustered according to a combination of the absence/presence of pigment spots and furrows. Participants had relatively the same levels of BIS/BAS in all four iris types. Behavioral inhibition and activation are relatively stable temperamental precursors different from DM.

The correlation analysis within the similarity measure (Clatworthy et al., 2005) showed a negative association between pigment spots and dispositional mindfulness. More mindful participants had no pigment spots on their irises. No correlations were observed between BIS/BAS and iris characteristics. Finally, pigment spots and furrows were not interrelated, which is inconsistent with previous findings (Sturm and Larsson, 2009).

From a psychological perspective, the findings may be inspirative for exploring the association between iris structures and DM. From the biological perspective, the results may not be surprising as there are indications that brain and eye structures are connected. For example, the brain/eye tissue loss hypothesis has been suggested in the original study examining eye structures and personality (Larsson et al., 2007). The retinal structure may reflect changes in the brain tissue. In other words, retinal change could be a readily accessible marker of structural and functional brain integrity (Phadikar et al., 2017). Iris structures investigated in the current study, pigment spots and contraction furrows, relate to the eye tissues’ thickness. Thinning of the iris positively correlates with iris pigmentation (Sturm and Larsson, 2009). Mindful participants in the current sample had many furrows and thus thicker irises (Chua et al., 2017). That means they could have a thicker brain tissue or stronger brain integrity (Phadikar et al., 2017), which may lead to better concentration and regulation of everyday experience. The findings allow more credible research regarding personality dispositions, self-regulation, and eye structures. Self-regulation and the brain are interrelated (Modinos et al., 2010; Ventura et al., 2019), and this connection may extend to the physiology of the eye. Indeed, research must take natural variability into account and should proceed with caution.

### 4.1. Future research

The current research findings also align with previous suggestions that the development of pigment spots is partly regulated by neurotransmitters produced by the autonomic nervous system (Hu, 2000; Hu et al., 2000). Neurotransmitters are crucial for the function of complex neural systems. For example, dopamine plays a role in the reward system, motivation, and emotional arousal. Serotonin is associated with mood (Loula and Monteiro, 2022) and peripheral tissues (Lv and Liu, 2017). Neurotransmitters relate to human personality and dispositional traits (Delvecchio et al., 2016). A biological pathway responsible for pigments spots formation and neurotransmitters might be the same. As the iris structures are moderate to highly heritable, the current findings also have implications for biologists. Specifically, the genetic variants responsible for pigmentation and green/brown eyes might be studied in the context of personality and neurotransmitters. Future research can also examine personality-related genes (Zwir et al., 2020), eye color, pigmentation, and tissue formation, suggesting an interdisciplinary approach involving cooperation between psychologists and biology researchers. For example, genes already associated with normal neuronal development (Larsson et al., 2011), eye color, pigmentation, and tissue formation (Sulem et al., 2008) might be investigated in relationship with DM. Our findings indicate that iris pigment spots might be examined further in the context of personality dispositions and abilities that relate to concentration, attention, or regulation of negative affect.

The longitudinal design of examining the connection between the eye iris and DM may have been enriching. Future research can also explore whether people with the absence of pigment spots and the presence of contraction furrows conduct DM techniques faster/easier. On the other hand, future research can examine how easy/hard it is to train people with furrows and pigment spots in DM techniques. Future research should also consider other relevant eye characteristics related to pigmentation (e.g., pigmented rings) and examine possible iris structure clusters and individual differences. Finally, research involving twin studies might be beneficial.

### 4.2. Limitations

There are several limitations concerning the results of the current study. The first limitation is the uneven sex (70% were women) and one race of participants, white university students. The second potential limitation is the incomplete complexity of the eye characteristics used in the current analysis. Only two iris characteristics (furrows and pigment spots) were analyzed. However, more relevant biological characteristics exist in the eye (e.g., Wolfflin nodules, crypts, and pigmented rings). Combining more iris characteristics can create more or different iris structure clusters. People with medium-sized contraction furrows (between 1/4 and 8/10) or five and more pigment spots were absent in the sample. Also, neither DM nor BIS/BAS have been previously investigated in the context of possible iris biomarkers. Only the left eye of the participants was analyzed.

### 4.3. Conclusion

The study yielded interesting results regarding iris pigmentation and self-regulation of personality. Cluster analysis of iris characteristics (presence/absence of pigment spots and extent of contraction furrows) converged at a four-cluster solution of iris structure. Cluster type without pigment spots and a large extent of contraction furrows had the highest level of dispositional mindfulness. Contrary, cluster type with the presence of pigment spots and large contraction furrows showed the lowest level of dispositional mindfulness. The other personality dispositions - behavioral activation and inhibition systems did not differ according to iris pigment spots and contraction furrows. The results align with previous research indicating that iris pigmentation may be linked to personality dispositions.

## Data availability statement

The raw data supporting the conclusions of this article will be made available by the authors, without undue reservation.

## Ethics statement

The studies involving human participants were reviewed and approved by Faculty of Social and Economic Sciences, Comenius University in Bratislava. The patients/participants provided their written informed consent to participate in this study. Written informed consent was obtained from the individual(s) for the publication of any potentially identifiable images or data included in this article.

## Author contributions

MV drafted the initial manuscript, conducted literature searches, and conceptualized the study. EL designed the methods, performed the statistical analysis, and supervised the manuscript. MV and EL edited the manuscript. All authors contributed to the article and approved the submitted version.

## Funding

The Scientific Grant Agency of the Ministry of Education, Science, Research and Sports of the Slovak Republic and the Slovak Academy of Sciences supported the manuscript with grant VEGA 1/0075/19. The funding agency had no role in the design of the study and collection, analysis, and interpretation of data and in writing the manuscript.

## Conflict of interest

The authors declare that the research was conducted in the absence of any commercial or financial relationships that could be construed as a potential conflict of interest.

## Publisher’s note

All claims expressed in this article are solely those of the authors and do not necessarily represent those of their affiliated organizations, or those of the publisher, the editors and the reviewers. Any product that may be evaluated in this article, or claim that may be made by its manufacturer, is not guaranteed or endorsed by the publisher.
